# Variation in *APOL1* Contributes to Ancestry-Level Differences in HDLc-Kidney Function Association

**DOI:** 10.1155/2012/748984

**Published:** 2012-09-02

**Authors:** Amy Rebecca Bentley, Ayo P. Doumatey, Guanjie Chen, Hanxia Huang, Jie Zhou, Daniel Shriner, CongQing Jiang, Zhenjian Zhang, Guozheng Liu, Olufemi Fasanmade, Thomas Johnson, Johnnie Oli, Godfrey Okafor, Benjamin A. Eghan, Kofi Agyenim-Boateng, Jokotade Adeleye, Williams Balogun, Clement Adebamowo, Albert Amoah, Joseph Acheampong, Adebowale Adeyemo, Charles N. Rotimi

**Affiliations:** ^1^Center for Research on Genomics and Global Health, National Human Genome Research Institute, National Institutes of Health, Bethesda, MD 20892-5635, USA; ^2^Suizhou Central Hospital, Suizhou, Hubei 441300, China; ^3^Department of Medicine, University of Lagos, Lagos 101017, Nigeria; ^4^Department of Medicine, University of Nigeria Teaching Hospital, Enugu 400001, Nigeria; ^5^Department of Medicine, University of Science and Technology, Kumasi, Ghana; ^6^Department of Medicine, University of Ibadan, Ibadan 200284, Nigeria; ^7^Department of Medicine and Therapeutics, University of Ghana Medical School, Accra, Ghana

## Abstract

Low levels of high-density cholesterol (HDLc) accompany chronic kidney disease, but the association between HDLc and the estimated glomerular filtration rate (eGFR) in the general population is unclear. We investigated the HDLc-eGFR association in nondiabetic Han Chinese (HC, *n* = 1100), West Africans (WA, *n* = 1497), and African Americans (AA, *n* = 1539). There were significant differences by ancestry: HDLc was positively associated with eGFR in HC (*β* = 0.13, *P* < 0.0001), but negatively associated among African ancestry populations (WA: −0.19, *P* < 0.0001; AA: −0.09, *P* = 0.02). These differences were also seen in nationally-representative NHANES data (among European Americans: 0.09, *P* = 0.005; among African Americans −0.14, *P* = 0.03). To further explore the findings in African ancestry populations, we investigated the role of an African ancestry-specific nephropathy risk variant, rs73885319, in the gene encoding HDL-associated APOL1. Among AA, an inverse HDLc-eGFR association was observed only with the risk genotype (−0.38 versus 0.001; *P* = 0.03). This interaction was not seen in WA. In summary, counter to expectation, an inverse HDLc-eGFR association was observed among those of African ancestry. Given the *APOL1* × HDLc interaction among AA, genetic factors may contribute to this paradoxical association. Notably, these findings suggest that the unexplained mechanism by which *APOL1* affects kidney-disease risk may involve HDLc.

## 1. Introduction

Dyslipidemia is a known correlate of chronic kidney disease (CKD), with increased triglycerides (TG) and decreased high-density lipoprotein cholesterol (HDLc) consistently observed [1, 2]. The relationship between lipids and kidney function in the general population, however, is less clear. As the response between glomerular and vascular cells to dyslipidemia is similar [3], an atherosclerosis-like mechanism of damage has been proposed in renal insufficiency [2, 4] and has been supported by animal models [5]. Dyslipidemia may contribute to the worsening of kidney function in the general population. While CKD prevention certainly motivates further elucidation of this relationship, kidney function monitoring has implications beyond disease risk. A common kidney function measure, estimated glomerular filtration rate (eGFR), is associated with cardiovascular disease and all-cause mortality [6–9] even with mild-to-moderate loss in kidney function [6, 7].

There are consistent reports of an inverse association between TG [10–14] and, to a lesser extent, total cholesterol [14, 15] with kidney function. The HDLc association, however, is less clear. A positive association between HDLc and kidney function has been observed [10, 12, 14–16] consistent with the atherosclerosis-like model; however, recent reports have found an inverse association between HDLc and kidney function [11, 17] although the biological mechanism for such a relationship is unknown.

Given the variety of methods used in projects that provide evidence on this relationship, it is difficult to ascertain whether such variability in association is a consequence of real population-level differences, specific kidney function measures considered, or analytical strategies. Therefore, we evaluated the relationship between HDLc and kidney function in different populations purposefully designed to have consistent data and biological specimen collection and analyzed identically.

Consideration of diverse populations is especially relevant in describing the HDLc-kidney function association for several reasons. First, the rates of CKD (and its progression to end-stage renal disease (ESRD) vary significantly by population and ancestry, for reasons that are unclear. For instance, in the US, African Americans have a nearly 3-fold higher risk of CKD [18], and both African and Asian Americans have higher rates of ESRD than European Americans (3.7-fold and 1.3-fold higher, resp.) [19]. African Americans have an approximately 5 times faster rate of progression from renal insufficiency to ESRD than European Americans [20]. Another motivation for investigating this relationship in diverse populations is the variation in lipid profiles among those with different ancestral backgrounds. African-ancestry populations, in particular, have significantly lower HDLc, TC, and TG distributions than their European-ancestry counterparts. The reason for these differences is unknown, but may imply a different relationship between lipids and disease. The existence of such a difference is supported by the “under-identification” of Africans and African Americans by the metabolic syndrome's lipid criteria: hypertension, type 2 diabetes, and cardiovascular disease risk remains high in these populations in the presence of “normal” values of TG [21, 22].

The recent discovery of missense variants in the *Apolipoprotein L1* gene (*APOL1*) that increase kidney disease risk [23] among African Americans makes the understanding of the HDLc-kidney function association very timely. Variants in the gene encoding this HDLc-associated lipoprotein are present in relatively high frequency in African ancestry populations, and are thought to have increased in distribution because of a protective advantage against a severe form of African sleeping sickness. The mechanism by which this variant influences kidney disease risk and, according to a recent report, allograft survival after kidney transplantation [24], has not yet been elucidated. We investigated the association between serum HDLc and eGFR in three independent epidemiological studies of West Africans, African Americans, and Han Chinese. Among African Americans and West Africans, the role of the nephropathy-associated *APOL1* variant, rs73885319, was also assessed.

## 2. Methods

### 2.1. Study Participants

Ethical approval for each study was obtained from the responsible Institutional Review Board (IRB) at each study site/institution, Howard University, and the National Institutes of Health. All subjects provided written informed consent for the collection of samples and subsequent analysis. This study was conducted according to the principles expressed in the Declaration of Helsinki.

Participants originated from three separate epidemiological studies: the Africa America Diabetes Mellitus (AADM) Study, the Howard University Family Study (HUFS), and the China America Diabetes Mellitus study (CADM). Further details of AADM [25] and HUFS [26] have been previously published. Briefly, AADM is a large-scale case-control study designed to explore the genetic and environmental determinants of type 2 diabetes (T2D) in West Africa. HUFS is population-based study of families and unrelated African Americans enrolled from the Washington, DC metropolitan area. CADM is a large-scale genetic epidemiology study designed to investigate the genetic and environmental determinants of T2D in unrelated Han Chinese from the Suizhou area in China. Participants completed questionnaires that were the same as those used in AADM and HUFS (with appropriate cultural adaptations) and participated in clinic visits. T2D cases, defined as being on physician-prescribed diabetes treatment or with fasting blood glucose ≥126 mg/dl on more than one occasion, were excluded from the present investigations.

There were 4574 non-T2D participants across the three studies. Of these, 381 participants were excluded for missing or extreme serum lipid measurements. Additional exclusions were made for missing covariates (BMI, *n* = 19; smoking  status, *n* = 32; alcohol use, *n* = 6). After exclusions, 4136 participants were considered in the main analysis. The main findings were also investigated in the nationally-representative National Health and Nutrition Examination Survey (NHANES) for years 2007-2008, and 2003-2004 [27, 28] (2005-2006 data was not included given observed undesirable bias for the HDLc assay [29]). After exclusions, 623 African Americans (NHAA) and 1709 European Americans (NHEA) were included (participants from both survey years were analyzed together).

### 2.2. Laboratory

Serum lipids (TG, TC, and HDLc) were determined enzymatically using methods standardized to in-house and other appropriate reference methods (e.g., CDC reference methods for HDLc, Isotope dilution mass spectrometry (ID-MS) for TC and TG). HDLc was assayed using homogeneous enzymatic colorimetric assays in all cohorts (COBAS Integra 400 Roche Diagnostics, IN, USA (WA, AA); AEROSET C8000, Abbott Diagnostics, China (HC), and Roche/Hitachi Modular P Chemistry Analyzer, Roche Diagnostics, IN, USA (NHANES). LDLc was calculated (using the Friedewald equation) [30].

For WA, AA, and NHANES, serum creatinine levels were determined using a Jaffé reaction without deproteinization on either a COBAS Integra 400 analyzer (Roche Diagnostics, IN, USA; WA and AA) or a Beckman Synchron Analyzer (Beckman Coulter Inc., CA, USA; NHANES). In HC, serum creatinine was assayed on AEROSET C8000 (Abbott Diagnostics, China) using an enzymatic reaction. Methods comparison conducted by the manufacturers shows that the two methods yielded acceptable correlation (*r* ~ 0.99: reference, http://www.accessdata.fda.gov/cdrh_docs/pdf7/K073634.pdf). These methods have been standardized against ID/MS and primary reference material (SRM 914).

### 2.3. Genotyping

To further evaluate results, we genotyped a major kidney disease *apolipoprotein L1* (*APOL1*) risk variant (rs73885319) in 1296 African Americans (HUFS) and 1095 West Africans (AADM). Genotyping was performed using the iPLEX Gold assay on the MassArray platform (Sequenom, San Diego, CA) as previously described [31]. Briefly, the PCR and extension primers were designed using MassArray designer Software, the sequence information was imported from public databases for catalogued SNPs. This platform uses a single-base primer extension chemistry with MALDI-TOF MS technology for detection. The average call rate for this SNP was 98.1% in HUFS and 97.2% in AADM. Among successfully typed individuals, genotype frequencies were in HWE (*P* > 0.05) and concordance of duplicate genotypes was >99.9%.

### 2.4. Statistical Analysis

We used as a measure of renal function the estimated glomerular filtration rate (eGFR), which was calculated according to race- and gender-specific Chronic Kidney Disease Collaboration equations [32]. Variables that were not normally distributed were transformed (BMI and all lipid variables). Alcohol intake was dichotomized into self-report of some versus no regular consumption. Smoking was represented categorically (smoker, ex-smoker, and nonsmoker).

Given multicollinearity between serum lipid measures and between these and BMI, these variables were mean-centered and normalized. No multicollinearity was observed among the transformed variables. The relationship between eGFR and predictors was analyzed using population-stratified linear regression models adjusted for age, age^2^, sex, BMI, smoking, LDLc and TG (included to isolate an association of HDLc, as opposed to a correlated serum lipid constituent), and alcohol use. For those studies with related participants (HUFS and AADM), an adjustment for the random effect of family was included. To formally evaluate differences in the association between HDLc and eGFR by population, all studies were included in the same model with an HDLc × study interaction term.

Consistent with published findings [33], *APOL1* rs73885319 was coded recessively (GG, referred to throughout as the “nephropathy risk genotype” versus GA/AA). The associations of genotype with HDLc and with eGFR were determined in linear mixed models adjusted for age, age^2^, and sex, with random clustering for family. For African Americans, principal component analysis to assess population structure was conducted using EIGENSOFT [34], and the first principal component, representing the proportion of African ancestry, was retained (as reported previously [35] ), and included in all models of rs73885319. To evaluate the interaction between the *APOL1* variant and HDLc, an rs73885319 x HDLc product term was added. All analyses were conducted using SAS 9.2 (SAS Institute, Cary, NC). Data was represented graphically using R (http://www.r-project.org/).

## 3. Results

A total of 4,316 participants met the criteria for inclusion (Table 1): 1,497 AADM participants (West Africans: WA); 1,539 HUFS participants (African Americans: AA); 1,100 CADM participants (Han Chinese: HC). WA had the highest eGFR (*P* < 0.0001 versus HC; *P* = 0.0006 versus AA). Notable differences in serum lipids between the populations were observed. Total cholesterol was much lower among WA than among AA or HC (*P* < 0.0001), reflecting a lower mean HDLc (*P* < 0.0001). Triglycerides were highly variable among the three studies, with WA having 15 mg/dl lower mean concentration than AA and nearly 50 mg/dl lower mean concentrations than HC (*P *< 0.0001 for all comparisons). Lower mean TG levels have been consistently observed in African- compared to European- or Asian-ancestry populations [36–42].

**Figure 1:**
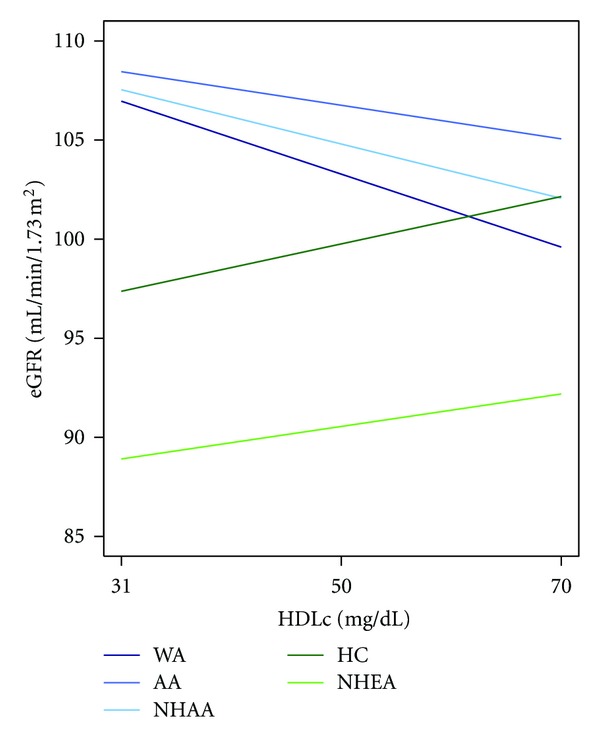
Association between HDLc and eGFR in Diverse Populations. The association of HDLc and eGFR in West Africans (WA), African Americans (from Howard University Family Study, AA), African Americans (from National Health and Nutrition Examination Survey, NHAA), Han Chinese (HC), European Americans (NHEA). Linear models adjusted for age, age^2^, sex, BMI, triglycerides, LDLc, alcohol use, and smoking (and random effect of family for WA and AA).

The association between HDLc and eGFR differed between studies (Figure 1). Most notably, while HDLc was associated with eGFR in each study, the direction of the association was different, with inverse association among WA ( *β* −0.19, *P* < 0.0001) for and AA ( *β* −0.09, *P* = 0.02) and positive association among HC ( *β* 0.13, *P *< 0.0001).

As expected, when all studies were included in a single model, an HDLc × study interaction term was highly statistically significant (*P* < 0.0001). This remained true when only two studies were considered at a time (WA versus AA (*P* = 0.001), WA versus HC (*P* < 0.0001), and AA versus HC (*P* < 0.0001)).

To determine the generalizability of the HDLc findings, analyses were run in European Americans and African Americans from NHANES (NHEA and NHAA, resp.). These findings supported the results found in the original analysis: HDLc was positively associated with eGFR among NHEA (*β*: 0.09, *P* = 0.005) and negatively associated with eGFR among NHAA (*β*: −0.14, P = 0.03).

To further evaluate the unexpected, but consistent, difference in association by African ancestry, the relationship between rs73885319, a SNP that has been associated with kidney disease among African Americans, was investigated in the AA and WA. The frequency of the nephropathy-associated allele (GG) was 0.21 and 0.38 in AA and WA, respectively.

**Figure 2:**
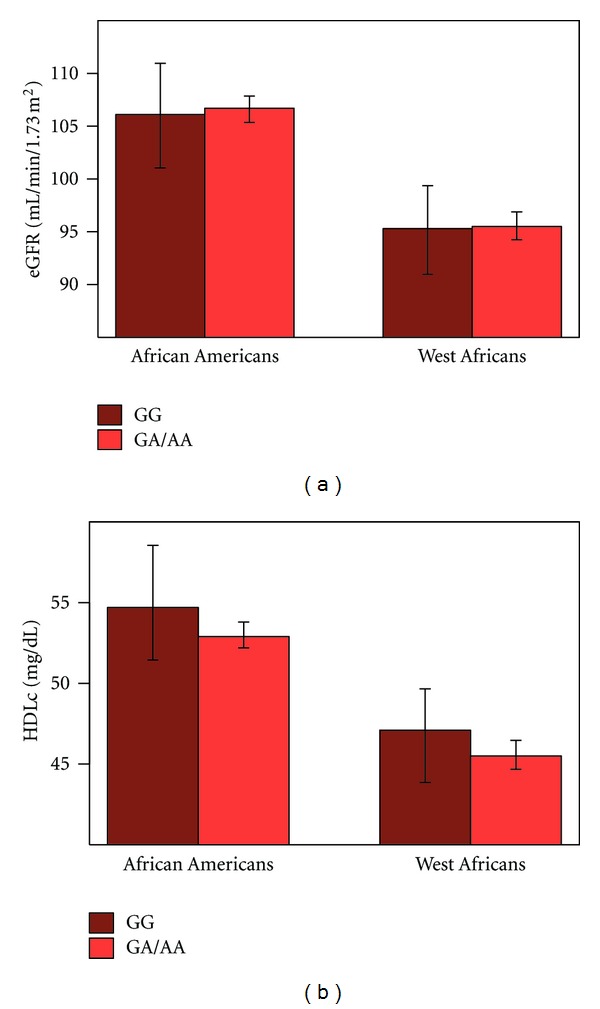
Mean HDLc and eGFR by rs73885319 Genotype among African Americans and West AfricansMean HDLc and eGFR by rs73885319 genotype (GG = nephropathy risk genotype), as estimated from linear mixed effect models adjusting for age, age^2^, sex, and overall proportion of admixture (African Americans only), with random clustering for family. There were no statistically significant differences in HDLc or eGFR by genotype.

There was no evidence of an association between the variant and eGFR in either population (Figure 2). Although no association was observed between the variant and HDLc in the population as a whole, among women, there was some evidence for a difference in HDLc among AA women by genotype (5.3 mg/dl higher with the risk genotype), although this did not remain statistically significant after adjustment for multiple tests (*P*_adjusted_ = 0.08). A formal test for a gender × rs73885319 interaction was not statistically significant (*P* = 0.13). There was no evidence of a difference by gender for either trait among the WA.

**Figure 3:**
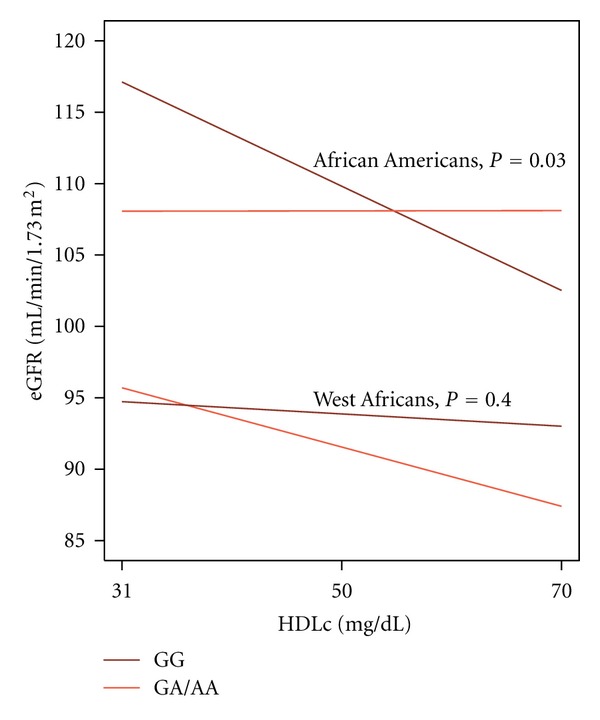
Association between HDLc and eGFR among African Americans and West Africans by rs73885319 Genotype. The modification of the association between HDLc and eGFR by rs73885319 genotype (GG = nephropathy risk genotype), as estimated from a linear mixed model of HDLc × rs73885319, adjusting for HDLc rs73885319, age, age^2^, sex, and overall proportion of admixture (African Americans only), with random clustering for family. Among those with the nephropathy risk genotype, the association was much more negative than among those without this genotype (*β*: −0.38 versus 0.001, *P*_interaction_ = 0.03).

Among AA participants, a statistically significant interaction between the variant and HDLc was observed: the inverse association between HDLc and eGFR was larger among those with the risk genotype compared to those without (*β*: −0.38 versus 0.001, *P*_interaction_ = 0.03; Figure 3). The results were unchanged with the addition of BMI, LDLc, and TG to the model. This interaction was not observed in WA (*P*_interaction_ = 0.4).

### 3.1. Sensitivity Analyses

To address the potential concern that diseased participants drove the observed findings (as no exclusion was made for current chronic kidney disease) a sensitivity analysis was conducted excluding those with eGFR < 60 mL/min/1.73 m^2^: neither the direction nor statistical significance of the HDL-eGFR associations were altered. Also, there is evidence that the equation used to estimate GFR [32] with only two levels for race is not accurate for Asians or African Blacks [43]. When we used an equation that included a distinct Asian coefficient [43] for HC and eliminated the “Black” coefficient for WA (coefficient found to produce more bias than omitting among South African Blacks [43]), the HDL-eGFR findings were unchanged. Similarly, no difference was observed when using the MDRD equation to estimate GFR instead of the CKD-EPI equation [44]. Lipid-lowering medication may affect both GFR and HDL concentration. While the use of these drugs is virtually nonexistent among the West Africans (AADM) and unknown in the Chinese (CADM), ~4% of African Americans (HUFS) participants used these drugs. When analyses were limited to the subset of African Americans with lipid-lowering medication use data (78% of participants), the inclusion of this term in models had no effect on the size, direction, or statistical significance of the associations of interest.

Although the HDL-eGFR results from the NHANES AA, which did not include relatives, were consistent with what was observed among WA and HUFS AA, which did include relatives, we conducted a sensitivity analysis to investigate the possibility that the adjustment for relatedness in our models was insufficient to remove a potential bias. When only unrelated participants were included, the association between HDL and eGFR was similar and remained statistically significant. When the analysis of the rs73885319 interaction in HUFS was limited to unrelated participants, the effect size was similar, though the *P-*value was increased (*P* = 0.4). The consistent effect size and diminished statistical significance suggest that this difference is due to a reduction in sample size and not bias, a conclusion further supported by the similar results obtained in rerunning analyses in similarly-sized random subsets of the related dataset. As such, there is no evidence from which to infer bias based on the inclusion of related individuals in two of the samples evaluated.

Additionally, no change in the observed results was found with adjustment for hypertension, hypertension treatment, or CRP (CRP not available in AADM or CADM). The use of categorical serum lipid variables did not provide additional insights, though the results were consistent with those observed in the analysis with continuous variables. No consistent patterns of association were observed when the analyses were repeated within strata of serum lipids to further describe the relationships.

To address potential concern that ethnicity-specific bias in the eGFR estimating equations could have resulted in an apparently different association by ancestry, we redid the analysis using serum creatinine as the outcome. The results were consistent with the reported observations (an inverse HDLc-eGFR association in non-African ancestry groups and a positive association in the African ancestry groups). 

## 4. Discussion

In this investigation of West African (WA), African American (AA), and Han Chinese (HC) adults, the association between kidney function and HDLc varied dramatically by ancestry: a positive association was observed among HC, and an inverse association was found among the two African-ancestry populations. A known nephropathy-associated variant (rs73885319) in the *APOL1* gene, which encodes an HDL-associated apolipoprotein, was investigated among AA. Among AA women, higher HDLc was observed among those with the nephropathy-associated genotype. Further, among African American men and women, the inverse association between HDLc and eGFR was only observed in those with the risk genotype.

The inconsistency in the direction of the HDLc-eGFR association in WA and AA compared to HC is an important contribution to the current conflicting literature. This variation, found in data that was consistently collected and identically analyzed for three separate epidemiological studies, suggests that the relationship varies at the population level. In support of these findings, a positive association was found in a nationally representative sample of European Americans (NHEA), while an inverse association was observed in a similar nationally representative sample of African Americans (NHAA). In agreement with our findings in non-African ancestry populations, low HDL was associated with increased incidence of CKD among Japanese [14] and with elevated creatinine at followup among primarily European Americans [15]. In contrast, in Dutch adults, HDLc and apolipoprotein A-1 were inversely associated with kidney function [17]. In the only prior study of Africans [11], the results supported our findings in African ancestry populations: HDLc was inversely associated with kidney function in Black South Africans. Two multiethnic studies have evaluated HDLc-kidney function, and both observed a positive relationship [10, 12] (only in an unadjusted model [12]). Neither of these studies, however, reported evaluation of a race/ethnicity interaction or stratified analysis. In fact, some studies including multiple ethnicities simply adjust for “race,” without reporting a formal evaluation of the different association by ethnicity (i.e., by testing an interaction term). To demonstrate the importance of performing these detailed analyses, we evaluated the HC and AA together, with an adjustment for ethnicity (and no interaction term for ethnicity and HDLc): there was no association of HDLc and eGFR (*P* = 0.2). Similarly, when all three populations were considered together, there was an inverse HDLc-eGFR association (*P* < 0.0001), despite the strong, statistically significant positive association observed in the HC alone. Without evaluating the interaction term, these data would have led us to conclude that either there was no association of HDLc and eGFR (HC and AA) or that the association could be described as a strong, inverse association between HDLc and eGFR (HC, AA, and WA), conclusions that are not appropriate given our analysis.

While a positive association of HDLc and eGFR seen in non-African ancestry groups is consistent with the expectation that higher HDLc is associated with a variety of favorable health outcomes, the inverse association observed among WA and two AA populations was not expected. One possibility is that genetically determined characteristics of the HDL particle or its associated proteins differ in those of African versus non-African ancestry in ways that would not be detectable in comparisons of HDLc concentration. For instance, variation may exist in the antioxidant or anti-inflammatory properties of HDL or in the size distribution of its particles in a way that is relevant for kidney function. It has been reported that those of African ancestry typically have a more favorable size distribution of lipid particles than those of non-African ancestry [45–47]. Although it is unclear how HDL particle size could influence kidney function, these known differences in a characteristic of the HDL particle by African ancestry is worthy of consideration given our findings. Unfortunately, we did not have the data available to test these hypotheses.

Another possibility is that HDLc has only an indirect relationship with kidney function, but rather is a biomarker of other genetically-determined traits that are more proximally associated with kidney function, and that this trait (or the relationship between this trait and HDLc) differs dramatically by ancestry. Further support for this hypothesis comes from a recent discovery related to HDLc and risk of myocardial infarction (MI), a relevant disease model given that glomerular and vascular cells have been shown to respond similarly to dyslipidemia [3]. Although the protective association between HDLc and heart disease is well-known, genetic variants that raise HDLc were not associated with a decreased risk of MI in approximately 21,000 cases and 95,000 controls [48], highlighting that the biological mechanisms underlying this well-established association may require further investigation. Notably, the generally healthier lipid profile among African Americans does not translate into a lower prevalence of cardiovascular disease, as rates of both coronary heart disease and stroke among African Americans differ by only ~1% compared to European Americans [49]. It is also useful to consider the relation between serum lipids and insulin resistance (IR) in this context. Serum lipids, especially TG and HDLc, are associated with IR in European ancestry individuals, but growing evidence supports a differing or absent association in African ancestry individuals (reviewed in [50–53]). Along with these findings, our results suggest the need for increased attention to the role of HDLc in disease, with a particular focus on different ethnicities.

*APOL1* variants, including rs73885319, are thought to have risen to high frequency in African ancestry populations in areas of endemic trypanosomiasis, as these variants confer protection against a deadly form of African sleeping sickness (these variants are virtually absent in non-African-ancestry populations). Despite the protective role of *APOL1* variants in the context of infectious disease, these variants are deleterious in a chronic disease context: through unknown mechanisms, variants increase the risk of nephropathy in African Americans [33]. In this analysis, we show that a nephropathy-associated variant modifies the association between HDLc and eGFR in African Americans such that an inverse association was only observed among those with the risk genotype. It is difficult to speculate regarding the biological mechanism underlying the observed interaction given that the known strong association of this variant and nephropathy in African Americans has yet to be unraveled. How exactly variants in an HDL-associated protein that confer protection against African Sleeping Sickness affect kidney disease is unknown, but their discovery does suggest a connection between HDL and the kidney that has not been well explored. Notably, a recent study of African Americans reported a decreased concentration of medium HDL particles among those with *APOL1* risk variants [54]. As in our study, no difference in total HDLc or in eGFR by genotype was detected. If variant forms of *APOL1* do influence the subfraction distribution and either this alteration or another variant-provoked change perturbs kidney function, these findings may offer an explanation for our results. Other potential explanations include an effect of the variant on eGFR that leads to downstream changes in HDLc or that the variant separately affects these two quantities. Unraveling the biological mechanisms underlying the observed associations is beyond the scope of this study design, but further work in this area is urgently needed as insights into this relationship may have significant impact on screening and treatment of kidney disease in African Americans.

The reason for the lack of interaction in West Africans (despite their genetic similarity), is unclear, though there are numerous differences between these populations that could be further explored. The distributions of eGFR and HDLc are different in AA compared to WA, even after adjustment for known covariates (Figure 2), suggesting the possibility that the interaction is only apparent at a particular level of HDLc or eGFR. Additionally, there are differences in a wide range of parameters between AA and WA (for instance, diet, infectious disease exposure, smoking, and obesity), and one of these may provide the biological context requisite to produce the observed effect.

Of interest, there was evidence of a gender effect in this analysis. Specifically, among the African Americans, European Americans, and the Han Chinese, stronger associations were observed in women and the difference in the HDLc-eGFR association by *APOL1* genotype in African Americans was also stronger in women. While most previous studies do not report an evaluation of gender differences in HDL-kidney function, a stronger association was observed among women in two recent studies that observed an inverse association of HDLc and kidney function [11, 17]. A few relevant gender differences suggest avenues for further consideration. As a result of estrogen-signaled changes, greater adipose store, and increased free fatty acid mobilization, there is a two-fold greater entry of VLDL into women's circulation, with increased VLDL transport and removal leading to similar mean VLDL but higher HDLc concentrations [55]. This enhanced metabolism may result in a greater sensitivity to perturbations. Also, in addition to having a higher mean HDLc concentration, the proportion of HDLc that is present as large HDL particles is higher among women [46]. Although, again, it is unclear how a difference in particle size distribution may affect kidney function, it is noteworthy that this hypothesis also follows from the new finding of differential HDL subclass distribution by *APOL1 *genotype [54].

Population-level differences are subject to a wide array of potentially confounding factors, making a claim of differences by ancestry difficult to assert with confidence. Although there are dramatic differences in environmental exposures and lifestyle factors among the groups considered here, further consideration yields support for ancestry differences. First, all models were adjusted for smoking, alcohol use, and BMI; three factors that vary importantly among groups and could potentially affect both HDL and eGFR (although the limitations of adequately representing lifestyle characteristics from questionnaire data are acknowledged). Additionally, potentially confounding lifestyle and cultural factors are not similar between the African American and West African populations in which the HDLc-eGFR association is consistent. While smoking, for instance, is very high among African Americans, it is barely present in the West Africans. Obesity rates are likewise, very different between West Africans and African Americans, as are comorbidities, alcohol use, and lipid-lowering medication use (virtually nonexistent among West Africans). It is difficult to imagine a lifestyle factor that would be more highly correlated among African Americans and West Africans than between African Americans and European Americans, and could explain the observed pattern of association. Given the similar ancestry but different environmental exposures of African Americans and West Africans, the consistently observed inverse association between HDLc and eGFR supports the inference of a role of shared genetic factors.

Our investigation has some notable strengths: (1) the three studies were all designed by the same researchers, with the questionnaires in use intentionally designed to be comparable, insofar as possible given the different cultural context of the questions; (2) having both an African American population as well as a West African ancestral population provides an excellent opportunity to examine complex disease risk in the context of similar ancestral background and highly different lifestyle and cultural exposures. Some limitations of this study that may or may not directly influence the interpretation of the observed findings are worth discussing: (1) we made no adjustment for socioeconomic factors such as education or income as neither the values nor the implications of the values in the societal context were expected to be comparable between studies (level of education, e.g., has remarkably different impact on participants' healthcare use, diet, and lifestyle factors in the sites of the three studies); (2) the cross-sectional nature of this analysis makes it impossible to establish whether the observed associations result from an effect of reduced GFR on HDLc concentration or an effect of raised HDLc on eGFR; (3) HDL was not characterized in terms of proinflammatory or pro-oxidant capacity or particle size, characteristics which may be key in understanding the observed relationships. However, in the studied populations, the distribution of lifestyle characteristics that would be expected to influence oxidant or inflammatory status of HDL is not consistent with these being an explanation for the observed results: the rates of pro-oxidant factors (smoking and obesity) are high in the urban African American sample, smoking is also quite high in the Han Chinese (in whom a positive association was observed), and smoking rates are very low and BMI distribution more moderate in the West Africans (in whom the strongest inverse association was observed). Finally, replication in another sample of African Americans is necessary to confirm the observed interaction between rs73885319 and HDLc.

In summary, this analysis reports the association of serum lipids with kidney function in diverse populations. The direction of the association between HDLc and eGFR varies by ancestry, with a positive association observed in Han Chinese and European Americans, and an inverse association in West Africans and African Americans. While the factors behind these population-level differences are unknown, a nephropathy-associated variant in *APOL1* modifies the HDLc-eGFR association in African Americans with a strong inverse association observed in those with the risk genotype, while no association was observed among those without the risk genotype. Further work investigating the role of *APOL1* variants in kidney disease should explore the effects of *APOL1* on HDLc.

## Figures and Tables

**Table 1:** Participant characteristics by cohort^1^.

Cohort	West Africans (WA)		African Americans (AA)		Han Chinese (HC)	
	Men	Women	Men	Women	Men	Women
*N *	618 (41%)	879 (59%)	602 (39%)	937 (61%)	571 (52%)	529 (48%)
Age (yrs)	40.4 (15.7)	40.7 (14.2)	43.6 (13.0)	43.0 (13.7)	52.5 (9.8)	51.3 (9.0)
Current smoker (%)	49 (8%)	14 (2%)	368 (61%)	409 (44%)	200 (35%)	19 (4%)
Former smoker (%)	118 (19%)	12 (1%)	72 (12%)	108 (12%)	70 (12%)	0 (0%)
Regular alcohol intake (%)	428 (69%)	468 (53%)	480 (80%)	650 (69%)	455 (80%)	105 (20%)
BMI (kg/m^2^)	23.7 (4.2)	27.0 (6.4)	28.5 (7.3)	31.4 (8.8)	24.6 (3.3)	23.4 (2.8)
Hypertension (%)	204 (33%)	306 (35%)	247 (41%)	360 (38%)	266 (47%)	166 (31%)
Total cholesterol (mg/dL)^2^	169.0 (61.5)	182.5 (61.0)	182.0 (56.0)	188.5 (52.5)	187.5 (46.8)	185.6 (41.4)
HDL (mg/dL)^2^	39.0 (17.0)	42.0 (19.0)	48.0 (20.0)	54.0 (21.0)	52.6 (19.7)	53.5 (19.7)
Triglycerides (mg/dL)^2^	75.0 (40.0)	73.0 (41.0)	91.0 (61.0)	81.5 (49.5)	124.0 (97.4)	97.4 (61.1)
Creatinine (mg/dL)	1.06 (0.23)	0.80 (0.20)	1.04 (0.36)	0.84 (0.44)	0.87 (0.36)	0.63 (0.16)
eGFR (mL/min/1.73 m)	105.2 (26.1)	109.4 (27.3)	104.6 (20.9)	104.4 (24.4)	98.1 (13.5)	103.0 (14.2)

^1^Shown are means (standard deviations) or *N* (percentages), except where indicated.

^2^Median (Interquartile Range) reported due to non-normality of trait.
